# Higher Physical Activity Level Improves Leptin Concentrations in Spinal Cord Injury Subjects

**DOI:** 10.1155/2021/9415253

**Published:** 2021-09-28

**Authors:** Ana Paula B. Ramkrapes, Renata G. Duft, Ivan L. P. Bonfante, Keryma C. S. Mateus, Joice C. S. Trombeta, Bruno Rodrigues, Mara Patrícia T. Chacon-Mikahil, Ricardo A. Tanhoffer, Claudia R. Cavaglieri

**Affiliations:** ^1^Lab. of Exercise Physiology, School of Physical Education, University of Campinas (UNICAMP), Campinas SP, Brazil; ^2^Lab. of Cardiovascular Adaptation & Exercise, School of Physical Education, University of Campinas (UNICAMP), Campinas SP, Brazil; ^3^Lab. of Cellular Biology, Department of Physiology, Federal University of Parana (UFPR), Curitiba PR, Brazil

## Abstract

The present study was designed to compare the body composition and indicators of chronic inflammatory grade, such as leptin, adiponectin, and resistin concentrations in irregularly active and active SCI subjects. Thirty-two male subjects participated in this study. They were divided into three groups: able-bodied control irregularly active (control, *n* = 11), irregularly active with SCI (SCI-IA, *n* = 8), and physically active with SCI (SCI-PA, *n* = 13). The enzyme-linked immunosorbent assay (ELISA) assessed serum concentrations of leptin, adiponectin, and resistin. All volunteers performed the maximum oxygen uptake (VO_2max_) test, 24 h total energy expenditure (TEE), and body composition by skinfold thicknesses. Leptin concentrations were higher in the SCI-IA group when compared to the other groups, while no significant differences were found between the SCI-PA and control cohorts. In addition, no significant differences were found among groups for serum adiponectin and resistin concentrations either. The SCI-PA group showed significantly higher values for TEE and VO_2max_ when compared to the other groups. Percentages of body fat and circumference were decreased in the control and SCI-PA groups when compared to the SCI-IA cohort. Associations between leptin and cardiorespiratory capacity and anthropometric markers were also observed. Our findings highlight that the lack of physical activity in the SCI subjects leads to poor general physical fitness and higher levels of body adiposity, which may induce hyperleptinemia, an essential marker for cardiometabolic disorders.

## 1. Introduction

Spinal cord injury (SCI) may trigger countless physical and physiological dysfunctions, therefore increasing morbidity and mortality rates in this population, making it a public health concern [1, 2]. Mechanical dysfunction should be emphasized among these changes since it severely limits mobility, which translates into a significant reduction in total energy expenditure (TEE) [3].

Reduction in basal metabolic rate (BMR) after SCI may change the TEE, which accounts for between 65 and 80% of the total daily energy required for general people [4, 5]. This fall in TEE may contribute to a positive energy balance, which leads to changes in body composition of these individuals, reducing muscle mass and increasing white adipose tissue (WAT), particularly in the visceral region [5, 6]. Indeed, low muscle mass and high WAT are key risk factors for the onset of clinical conditions, such as type 2 diabetes mellitus (DM2) and cardiovascular disease, as they act as critical secretory sources of proinflammatory adipokines [6–8].

Individuals with SCI present high concentrations of proinflammatory adipokines, which are associated with the progression of a chronic inflammatory grade compared to controls without SCI [3, 8, 9]. Leptin is among the most abundant adipokines and regulates body adiposity by modulating food intake and energy expenditure, acting on the hypothalamus, and signaling information about energy stocks. It may inhibit caloric intake and stimulate energy expenditure through the sympathetic nervous system, thus playing an important defense mechanism against obesity [10–12]. These have been associated with the increased insulin resistance [13] found in obese people and DM2 obesity-associated individuals [14, 15]. Moreover, adiponectin is another common adipokine and plays a crucial role as an anti-inflammatory cytokine, demonstrating a strong correlation with body mass index (BMI) in individuals with and without SCI [16, 17].

In order to design strategies to reduce the onset and progression of chronic diseases in the population with SCI, recent studies have investigated the effect of exercise on inflammatory markers. Rosety-Rodriguez et al. [18] have shown that aerobic training reduces concentrations of proinflammatory adipokines and waist circumference. However, few studies have investigated the impact of physical activity levels and cardiometabolic markers in people with SCI. Thus, the present study was designed to compare the body composition and indicators of chronic inflammatory grade, such as leptin, adiponectin, and resistin concentrations in irregularly active and active SCI subjects.

## 2. Methods

### 2.1. Subjects

Thirty-two men with quadriplegia participated in the present study. As inclusion criteria of the study, the subjects should (1) have more than two years of injury and (2) perform maximum physical tests. As exclusion criteria, the subjects must not have cardiovascular diseases and diabetes mellitus and do not use hypertensive, anticoagulants, and anti-inflammatory drugs. All volunteers signed a free and informed consent form, and the Ethics and Research Committee of the University of Campinas approved this study (CAAE: 61607816.2.0000.5404).

The groups were divided based on the physical activity level questionnaire physical and leisure (Godin-Shephard Leisure-Time Physical Activity Questionnaire (GSLTPAQ) [19]). Thus, the subjects were allocated in control (irregularly active subjects without SCI), SCI-IA (SCI irregularly active subjects), and SCI-PA (SCI physically active subjects).

### 2.2. Study Design

**Figure 1:**
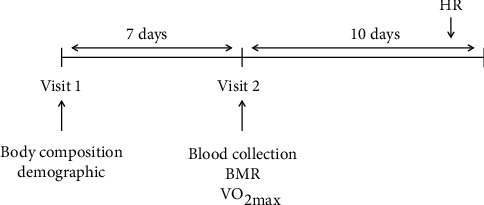
Experimental design. BMR: basal metabolic rate; VO_2max_: maximum oxygen uptake; HR: heart rate.

Participants visited two times the School of Physical Education at the University of Campinas. Demographic assessments and body composition were performed during the first visit. After seven days, the subjects returned for the second visit to collect blood samples and to assess the basal metabolic rate (BMR). For this procedure, subjects were instructed to fast 10 to 12 hours and avoid alcohol, physical exercise (for 48 hours), and caffeine for 24 hours. This evaluation occurred between 7:00 am and 10:30 am. Subsequently, the subjects ingested a breakfast, followed by an interval of 1 h and 30 min. Finally, they were submitted to the maximum oxygen uptake (VO_2max_) test and total energy expenditure (TEE) calculations and characterization of the groups. At the end of visit 2, the subjects received the equipment for heart rate measurement (POLAR®, model M400), and they should perform two heart rate (HR) collections (Figure 1).

### 2.3. Godin-Shephard Leisure-Time Physical Activity Questionnaire

On visit 1, participants answered the physical activity level questionnaire: Godin-Shephard Leisure-Time Physical Activity Questionnaire (GSLTPAQ) [19]. The protocol consists of answering how many times the subject performs physical activities during the week. Then, a formula was used that multiplies the number of activities performed at different intensities: weekly leisure activity score = (9x intense) + (5x moderate) + (3x light). For the division of the groups, the score was used as follows: (i) physically active: above 24 units; (ii) moderately active: 14 to 23 units; and (iii) insufficiently active: below 14 units. In the present study, the subjects were classified into spinal cord injury-irregularly active (SCI-IA, *N* = 8), spinal cord injury-physically active (SCI-PA, *N* = 13), and irregularly active (able-bodied) control (control, *N* = 11).

### 2.4. Body Composition

A digital electronic platform scale was used (LD 1050, Líder®) to get the value of body mass (kg). Body mass index (BMI) was estimated through the division of body mass in kg by the square of the height in meters: BMI = kg/m^2^ [20]. The skinfold model was used to assess body composition, collected using the Mitutoyo plicometer equipment (Cescorf®), on the subject's right side, by the same evaluator. Initially, Bulbulian et al.'s study [21] was used to estimate body density, and the Siri equation (1961) [20] was applied to estimate the percentage of fat (%Fat).

### 2.5. Blood Collection

All individuals were instructed not to consume caffeine and alcohol 24 hours before collection. The blood was subsequently centrifuged at 5000 rpm for 10 minutes. According to the specifications of the manufacturer, the serum concentrations of leptin, adiponectin, and resistin were determined in duplicates by enzyme-linked immunosorbent assay (ELISA) (Quantikine High Sensitivity Kit and DuoSet Kit, both by R&D Systems, Minneapolis, MN, USA). The assay range was as follows: 31–2000 pg/mL for leptin, 62.5–4000 pg/mL for adiponectin, and 39.0–2500 pg/mL for resistin.

### 2.6. Basal Metabolic Rate

To evaluate basal metabolic rate (BMR), a gas analyzer with breath-to-breath records was used (CPX, Medical Graphics, St. Paul, Minnesota, USA), performed after a 12-hour fasting period. Upon arriving at the laboratory, the subjects remained at rest, sitting in their wheelchair for 5 minutes. Then, they were transferred to a stretcher, without any physical effort, for the supine position for 15 minutes. After this period, the collection started and lasted for 30 minutes, simultaneously with the HR measurement, using the POLAR® monitor, used for TEE calculations set out below.

### 2.7. Maximum Oxygen Uptake (VO_2max_) Testing

In the arm ergometer (EB 4100, CEFISE), oxygen uptake was collected by a gas analyzer breath by breath (CPX, Medical Graphics, St. Paul, Minnesota, USA), and HR was measured using a POLAR®. A baseline collection of two minutes was initiated, and the testing protocol was started with a previous warm-up of 2 minutes, with a constant load of 0.13 watts (W) at speed between 50 and 60 rotations per minute. After a warm-up, the load was increased by 0.26 W each minute, in the same rotation, until the subject reached voluntary exhaustion [22, 23]. The test was considered maximum and ended when two or more of the following criteria were met: a plateau in oxygen uptake, a respiratory exchange rate (RER) greater than 1.1, the subject unable to maintain frequency between 50 and 60 rotations per minute, and the subject unable to continue the test. The maximum oxygen uptake (VO_2max_) was defined as the mean oxygen consumption values over the final 30 s of the test.

### 2.8. Total Energy Expenditure

The FLEX-HR method calculates energy expenditure based on HR data, using individual calibration from simple linear regression between HR and oxygen uptake (VO_2_) [24]. For the FLEX-HR calculation, the individual HR and VO_2_ collections were used under the following conditions: (a) in the supine position (BMR), (b) sitting position at rest, and (c) during the maximum oxygen uptake test on the arm ergometer, with incremental speed. Then, the FLEX-HR value was calculated, determined by the average between the highest HR values in the resting stages and the lowest HR value during exercise plus 10 b^-·min^.

To estimate TEE, HR collections were performed for 24 hours, recorded beat by beat. The subjects were instructed to use the HR monitor (POLAR®, M400), storing in a portable microcomputer. The HR data were downloaded to a computer, and the averages per minute (1440 minutes) were calculated. For each HR value identified as below the FLEX-HR, the corresponding VO_2_ value was used. For each HR value identified as above the FLEX-HR, the inclination of the HR/VO_2_ of each subject and the interception equation were determined. For each HR value measured, there was a corresponding VO_2_ value. Finally, the TEE was calculated by adding each subject's kcal/min values, totalizing 1440 minutes (24 hours) [25].

### 2.9. Physical Activity-Associated Energy Expenditure (PAEE)

The FLEX-HR method calculates energy expenditure in 24 hours. To measure the contribution of physical activity, the PAEE was estimated by the equation PAEE = (TEE × 0.90) − BMR, where 0.90 refers to the temperature of the thermal effect of food, with a constant fraction of 10%. Therefore, when removing energy expenditure due to the thermal effect of food and the BMR value, the remaining energy values refer to energy expenditure in physical activities that require more significant energy expenditure in addition to baseline values.

### 2.10. Statistical Analysis

Data analyses were performed using GraphPad Prism 8.0 and SPSS 25.0 software for Windows. The Shapiro-Wilk test was performed to test the normality of the data. For data considered parametric, an independent *t*-test was applied and Mann–Whitney for nonparametric variables. For the other features (age, BMI, and cytokines), ANOVA one-way test was applied, followed by Tukey post hoc for parametric data, and Kruskal-Wallis followed by Dunn's post hoc for nonparametric data. Pearson's correlation and simple linear regression were conducted to determine the correlation between leptin and the variables of characterization of the sample and fitness (body composition, TEE, and VO_2max_). The results are presented as mean and standard deviation (*M* ± SD). The *P* value equal to or less than 0.05 was considered statistically significant. The effect size was calculated using Hedge's *g* method (difference between the means of group 1 and group 2 divided by the pooled standard deviation and multiplied by a correction factor), being considered very small = *d* (0.1), small = *d* (0.2), medium = *d* (0.5), large = *d* (0.8), very large = *d* (1.2), and huge = *d* (2.0) effects.

## 3. Results

The profiles of the subjects are described in Table 1. No differences were found between the control, SCI-IA, and SCI-PA groups for age, BMI, and time since injury. The SCI-PA group showed significant differences in cardiorespiratory fitness for VO_2max_ (absolute and relative), HR_max_, TEE, GSLTPAQ, %Fat, and waist circumference (WC) compared to the SCI-IA group. However, no statistically significant difference was found for BMR data between groups.

**Figure 2:**
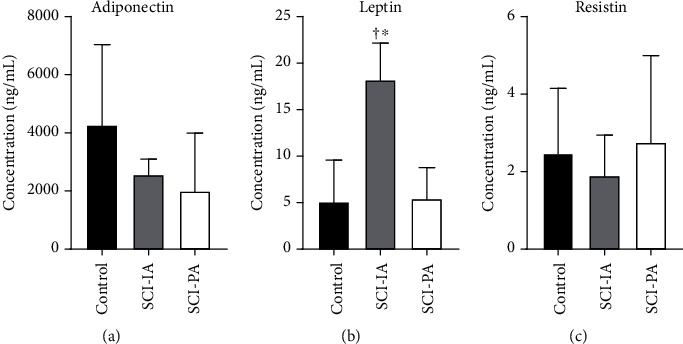
Adipokine levels in studied groups. (a) Adiponectin, (b) leptin, and (c) resistin concentrations for control (irregularly active subjects without spinal cord injury), SCI-IA (irregularly active SCI subjects (black bar)), and SCI-PA (physically active SCI subjects (grey bar)). ^∗^Significant difference with the SCI-IA group (*P* < 0.05). ^†^Significant difference with the control group (*P* < 0.05).

Figure 2 displays the adipokine levels in the studied groups. Adiponectin concentrations (Figure 2(a)) were similar between the control (4263.69 ± 2799.28 ng/mL), SCI-IA (2561.06 ± 565.27 ng/mL), and SCI-PA (1995.84 ± 2021.07 ng/mL) groups. Differences in leptin concentrations (Figure 2(b)) between the SCI-IA (18.17 ± 4.05 ng/mL) and control (5.06 ± 4.59 ng/mL) groups and between the SCI-IA and SCI-PA groups (5.43 ± 3.43 ng/mL) are shown. However, no differences were found between the SCI-PA and control groups. In addition, resistin levels (Figure 2(c)) were no different between the control (2.47 ± 1.69 *μ*g/mL), SCI-IA (1.89 ± 1.06 *μ*g/mL), and SCI-PA (2.75 ± 2.26 *μ*g/mL) groups.

**Figure 3:**
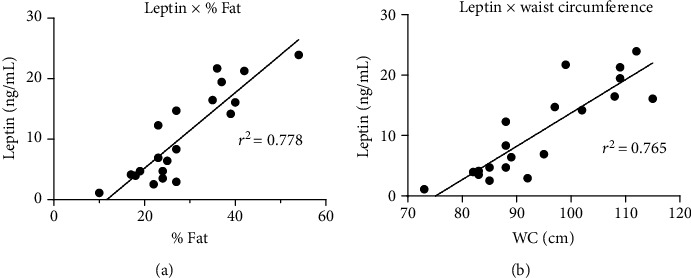
Linear regression between (a) leptin levels and %Fat and (b) leptin and waist circumference (WC), between the SCI-IA (*N* = 8) and SCI-PA (*N* = 13) groups.

Associations (simple linear regression) were observed between leptin levels and %Fat (Figure 3(a)) and between leptin and waist circumference (Figure 3(b)) for the SCI-IA and SCI-PA groups. Significant correlations were also observed between studied variables (Table 2), such as leptin levels and VO_2max_, waist circumference, and %Fat; TEE and BMR; HR_max_, VO_2max_ (mL/kg/min), and GSLTPAQ; VO_2max_ (mL/min) and HR_max_, BMR, and GSLTPAQ; HR_max_ and GSLTPAQ; waist circumference and %Fat; waist circumference and BMI and between %Fat and BMI.

Negative correlations were also found between adiponectin levels and VO_2max_ (mL/kg/min) and GSLTPAQ; leptin levels and VO_2max_ (mL/kg/min); VO_2max_ (mL/min), HR_max_, and GSLTPAQ; VO_2max_ (mL/kg/min) and waist circumference, %Fat, and BMI; and VO_2max_ (mL/min), waist circumference, and %Fat.

## 4. Discussion

Our main finding lies in the high levels of leptin concentrations observed in the insufficiently active SCI subjects compared with actives. Furthermore, associations between leptin and cardiorespiratory capacity and anthropometric markers were observed.

We found that leptin concentrations were increased in the SCI-IA group compared to the SCI-PA and control cohorts, suggesting that lower levels of physical activity may indicate a greater risk of cardiovascular disease in people with SCI. In addition to the adverse effects of sedentary conditions on leptin concentrations, previous studies have suggested that the sympathetic nervous system may be an essential regulator of leptin activity. Rayner et al. [10] have used alpha-methyl-p-tyrosine in an animal model, a substance capable of limiting the rate of norepinephrine synthesis, resulting in sympathetic tone reduction. This study demonstrated that administration of alpha-methyl-p-tyrosine led to hyperleptinemia. Several other studies have likewise demonstrated higher leptin concentrations in individuals with cervical SCI when compared to thoracolumbar SCI, suggesting that decentralization of the sympathetic nervous system may impair proper leptin regulation in these subjects [12, 26].

On the other hand, our study indicates that the SCI-IA group showed differences in leptin concentrations. Additionally, no significant differences in leptin concentrations between the SCI-PA group and the control group were observed. Therefore, the decentralization of the sympathetic nervous system in individuals with SCI at the cervical level does not seem to be the main factor for hyperleptinemia in this population. In this sense, we attempted to determine the potential contributing factors to such an increase in SCI-IA subjects.

Adipose tissue is considered a leptin-secreting organ, and its exacerbated increase contributes to augmented concentrations, which may lead to hyperleptinemia [8]. When performing a linear regression of the SCI-IA and SCI-PA groups, we found a strong correlation between leptin concentrations and fat mass percentage and leptin concentration and waist circumference. Similar data may be found in a study carried out by Jeon et al. [26], who demonstrated a positive correlation between leptin concentration and fat mass (kg). We also attempted to determine the contribution of body composition to leptin concentrations. Pearson's correlation was performed in all groups, and leptin concentration was positively correlated with the percentage of fat mass, waist circumference, and body mass index. In line with our findings, Wang et al. [12] and Jeon et al. [26] have reported a positive correlation between leptin concentration and both body mass index and adipose tissue in people with SCI, suggesting that the increase in adipose tissue may be a potential contributor to changes in leptin concentration in these individuals. This is mainly due to the increase in subclinical inflammation associated with excess body adiposity, leading to metabolic dysfunctions, such as decreased signaling of leptin receptors and the resulting resistance to leptin and hyperleptinemia [27].

Interestingly, when comparing the SCI-PA and control groups, there was no difference in the fat mass percentage, waist circumference, and leptin concentration, but the SCI-PA group had higher physical activity level scores in the used questionnaire. This finding shows that the practice of physical activity can contribute to the control of body adiposity in SCI and reduce its impact on the organism, reaching similar conditions to the non-SCI subjects.

The practice of physical activity has been proposed as a strategy to improve body composition and the inflammatory profile of the body. In this sense, we investigated the contribution of physical activity to leptin concentrations in subjects with SCI. Pearson's correlation showed negative correlations between leptin and indicators of physical activity level, although there was no direct correlation with total energy expenditure. A negative correlation was found between leptin and VO_2max_, maximum heart rate, and scores of physical activity questionnaire, suggesting that regular physical activity is associated with a reduction in leptin concentrations.

Comparing the levels of total energy expenditure and physical activity between the SCI groups, the SCI-PA group presented higher values of both than the SCI-IA cohort. Unlike total energy expenditure, no difference in basal metabolic rate was observed between the groups, corroborating other studies in this population [5, 6]. SCI-PA subjects were found to spend, on average, 600 kcal/day when compared to SCI-IA; as such, despite the absence of statistical significance, we cannot ignore the clinical relevance of such higher daily energy expenditure. That means roughly 20.000 kcal per month, enough energy to increase body adiposity and increased risk for pathological conditions, such as hyperleptinemia and its consequences.

In this sense, we demonstrated that the SCI-PA group had high health quality indicators, such as VO_2max_, compared to SCI-IA. These findings have also been observed by other researchers [28, 29], as they showed that systematized physical activity contributes to improvement in cardiorespiratory fitness in this population, which is related to the high quality of life.

Indeed, current evidence suggests cardiometabolic risk profile, such as abdominal fat distribution, glycemic control, and high lipid levels, as a critical factor in the onset of cardiovascular diseases and type 2 diabetes mellitus in SCI [28–30]. Cragg et al. [14] suggested that sedentary lifestyle habits may contribute to the progression towards diabetes in SCI, with a peak of development about two decades before the control cohorts. In this sense, our study suggests that the regular practice of physical activity for SCI is an effective strategy to reduce cardiometabolic risk. For Garshick et al. [2], cardiometabolic risk largely accounts for the high mortality rate in this population.

The main limitation of our study lies in the studied sample. This was due to the difficulty in finding subjects with SCI available to undergo the required assessments since they were physically inactive subjects and the university laboratory is located on the second floor, without an elevator. Another limitation is the lack of health quality evaluations in the control group due to the subjects' unavailability to perform them.

In summary, our findings show the importance of the regular practice of physical activity, as the lack of exercise in the SCI subjects leads to poor general physical fitness and high levels of body adiposity, which may induce hyperleptinemia an essential marker for cardiometabolic disorders.

## Figures and Tables

**Table 1:** Characteristics of participants.

Variables	Control (*n* = 10)	SCI-IA (*n* = 8)	SCI-PA (*n* = 13)	*P* value	ES
Description					
Age (years)	35.70 ± 8.73	38.50 ± 6.02	33.27 ± 5.62	0.286	
Injury time (years)	—	12.25 ± 6.73	12.36 ± 2.73	0.960	
Fitness					
VO_2max_ (mL/kg/min)	—	5.53 ± 2.32	13.15 ± 3.73^∗^	<0.001	2.25
VO_2max_ (mL/min)	—	460.10 ± 169.49	922.83 ± 263.82^∗^	<0.001	1.92
HR_max_ (bpm)	—	91.66 ± 24.23	127.25 ± 15.50^∗^	0.001	1.74
TEE (kcal/day)	—	1479.77 ± 350.74	2390.50 ± 1098.53^∗^	0.038	1.00
BMR (kcal/day)	—	1099.98 ± 192.26	1249.29 ± 226.31	0.095	0.77
GSLTPAQ (au)	6.60 ± 8.05	12.75 ± 8.54	41.46 ± 12.98^†∗^	<0.001	1.22
PAEE (kcal/day)	—	263.15 ± 247.79	909.29 ± 908.26	0.206	0.85
Body composition					
%Fat	19.80 ± 0.07	38.21 ± 8.47^†^	21.97 ± 5.13^∗^	<0.001	-2.34
WC (cm)	86.0 ± 10.93	105.25 ± 8.64^†^	86.67 ± 6.23^∗^	<0.001	-2.41
BMI (kg/m^2^)	24.20 ± 3.50	24.87 ± 2.10	21.80 ± 1.47^∗^	0.006	-1.90

Values presented as mean ± SD. ES: effect size; BMI: body mass index; VO_2max_: maximum oxygen uptake; HR_max_: maximum heart rate; TEE: total energy expenditure in 24 hours; BMR: basal metabolic rate; %Fat: percentage of fat; WC: waist circumference. ^∗^Significant difference with the SCI-IA group (*P* < 0.05); ^†^significant difference with the control group (*P* < 0.05).

**Table 2:** Pearson's correlation coefficients in control (irregularly active subjects without spinal cord injury), SCI-IA (irregularly active SCI subjects), and SCI-PA (physically active SCI subjects).

	Adiponectin (ng/mL)	Leptin (ng/mL)	Resistin (*μ*g/mL)	TEE (kcal/day)	BMR (kcal/day)	VO_2max_ (mL/kg/min)	VO_2max_ (mL/min)	HR_max_ (bpm)	WC (cm)	%Fat	BMI (kg/m^2^)	GSLTPAQ (au)
Adiponectin (ng/mL)	1	0.4	-0.1	-0.3	-0.1	-0.5^∗^	-0.3	-0.3	0.4	0.4	0.4	-0.6^†^
Leptin (ng/mL)	0.4	1	-0.1	-0.3	-0.2	-0.8^†^	-0.6^†^	-0.7^†^	0.9^†^	0.9^†^	0.8^†^	-0.7^†^
Resistin (*μ*g/mL)	-0.1	-0.1	1	-0.2	-0.2	0.0	0.1	0.0	0.0	-0.1	0.1	0.2
TEE (kcal/day)	-0.3	-0.3	-0.2	1	0.9^†^	0.5^∗^	0.6^†^	0.4	-0.3	-0.4	0.0	0.3
BMR (kcal/day)	-0.1	-0.2	-0.2	0.9^†^	1	0.4	0.5^∗^	0.3	-0.2	-0.3	0.1	0.2
VO_2max_ (mL/kg/min)	-0.5^∗^	-0.8^†^	0.0	0.5^∗^	0.4	1	0.9^†^	0.7^†^	-0.8^†^	-0.8^†^	-0.6^†^	0.7^†^
VO_2max_ (mL/min)	-0.3	-0.6^†^	0.1	0.6^†^	0.5^∗^	0.9^†^	1	0.6^†^	-0.7^†^	-0.7^†^	-0.4	0.5^∗^
HR_max_ (bpm)	-0.3	-0.7^†^	0.0	0.4	0.3	0.7^†^	0.6^†^	1	-0.9^†^	-0.8^†^	-0.8^†^	0.7^†^
WC (cm)	0.4	0.9^†^	0.0	-0.3	-0.2	-0.8^†^	-0.7^†^	-0.9^†^	1	0.9^†^	0.9^†^	-0.7^†^
%Fat	0.4	0.9^†^	-0.1	-0.4	-0.3	-0.8^†^	-0.7^†^	-0.8^†^	0.9^†^	1	0.7^†^	-0.7^†^
BMI (kg/m^2^)	0.4	0.8^†^	0.1	0.0	0.1	-0.6^†^	-0.4	-0.8^†^	0.9^†^	0.7^†^	1	-0.7^†^
GSLTPAQ (au)	-0.6^†^	-0.7^†^	0.2	0.3	0.2	0.7^†^	0.5^∗^	0.7^†^	-0.7^†^	-0.7^†^	-0.7^†^	1

BMI: body mass index; VO_2max_: maximum oxygen uptake; HR_max_: maximum heart rate; TEE: total energy expenditure in 24 hours; BMR: basal metabolic rate; %Fat: percentage of fat; WC: waist circumference. ^∗^Significant correlation coefficients (*P* < 0.05). ^†^Significant correlation coefficients (*P* < 0.001).

## Data Availability

The data can be found in the repository: 10.6084/m9.figshare.16678414.
